# Mining the key regulatory genes of chicken inosine 5′-monophosphate metabolism based on time series microarray data

**DOI:** 10.1186/s40104-015-0022-3

**Published:** 2015-05-23

**Authors:** Teng Ma, Lu Xu, Hongzhi Wang, Jing Chen, Lu Liu, Guobin Chang, Guohong Chen

**Affiliations:** Animal Genetic Resources Laboratory, College of Animal Science and Technology, Yangzhou University, 88 South of University Ave., Yangzhou, Jiangsu 225009 P. R. China

**Keywords:** Co-expression, Hub genes, IMP metabolism, Regulation network

## Abstract

IMP (inosine 5′-monophosphate) is a compound that enhances the flavor of poultry meat. IMP has become a new breeding trait to improve poultry meat quality. We tried to identify several potential regulatory genes, and construct their predicted regulatory relationships. Time series gene expression profiles of thigh muscle tissues of Rugao chicken, a famous indigenous breed in China, were performed for analysis of genes that are co-expressed or correlated with the concentration of IMP. We found 15 crucial co-expression genes, which are *Hspa2, Pten, Gabpa, Bpi, Mkl1, Srf, Cd34, Hspa4, Etv6, Bmpr2, Gde1, Igfbp5, Cd28, Pecam1 and Gja1,* that may directly or indirectly regulate IMP metabolism. Eventually, we computed the correlation coefficient between 19 IMP Genes and 15 CGs (15 co-expression genes), and we identified and constructed a predicted regulation network. In conclusion, variation of IMP concentration was primarily connected with the muscle development process. During this process, 15 CGs were identified that may have significant influence on IMP metabolism. In particular, *Bmpr2*, *Pten* and co-expression genes correlated with *Entpd8* might play important roles in regulating IMP de novo synthesis, decomposition and salvage synthesis.

**Electronic supplementary material:**

The online version of this article (doi:10.1186/s40104-015-0022-3) contains supplementary material, which is available to authorized users.

## Introduction

IMP (inosine 5′-monophosphate) is one of the key compounds that enhances the flavor of livestock and poultry meat [1, 2]. At present, IMP has been known that it has important effect on high-quality meat due to the improvement of water-holding capacity, physical, sensory properties of heat-induced gels from porcine meat and the growth and health of nursery pigs [3, 4].

IMP is an intermediate of purine metabolism [5]. Different measurement methods and storage conditions have significant influence on IMP content [6]. Gorostiaga *et al.* found in humans that after sport activities, IMP content rose significantly when phosphocreatine was reduced [7]. However, some chicken breeds have been proved that they have higher IMP content than that of other breeds [8–10]. The heritability of IMP content in animal muscle tissues varies from 0.23 to 0.6025 [11, 12]. Therefore, we believed that genetic factors have a large influence on IMP content between different breeds.

Different chicken breeds have different development rules, thus the muscle development may have a large effect on IMP metabolism. The IMP in the muscle of 42 days of age chicken is easier enriched than 21 days of age chicken [12]. Shubo [13] found that the IMP content in breast and thigh of AA commercial line at 8 and 13 wk of age (wk) was significantly higher than at 4 wk, but they all did not further investigate the genetic basis of the phenotype.

In order to explore the key regulation pathways influencing IMP metabolism, determining the whole genome expression profile has not been carried out yet. Although those IMP metabolism related genes such as *GARS*, *AIRS*, *GART*, *GPAT*, *AIRC* and *PURH* have been found that some genotypes of those genes is highly correlated with the IMP content and their expression patterns were detected as well [14–16], there probably are other crucial regulators we did not notice.

In this study, all experiments were performed in a relatively stable external environment. The co-expression or co-regulation networks and key regulators involved in IMP synthesis, decomposition and utilization were analyzed during the process of development of the thigh muscles of both female and male Rugao chicken. Rugao chicken is a famous Chinese indigenous poultry breed originating from the Yangtze River basin in Jiangsu province, has been widely researched in China because of the fresh and tender texture of its muscles and high stress resistance [17]. The aim of this study was to provide useful information regarding key regulators of chicken IMP metabolism and, subsequently, determine a crucial gene group that is considered to encompass the candidate genes for use in breeding to improve the quality of poultry meat.

## Material and methods

### Ethics statement

All experimental procedures were performed in accordance with the Administration Act of Experimental Animals using and care in Jiangsu Province (#115th Jiangsu Province Government Notice in 2008). All of the animal experimental operations were approved and guided by the Animal Care and Use Committee of Yangzhou University.

### Sample preparation

Experimental chicks were from a pure line of Rugao chicken from the Poultry Institute, Chinese Academy of Agricultural Sciences and were raised in individual cages in the closed house with an environment auto-control system from 2 to 12 wk. Food and water were given *ad libitum*.

All experimental procedures were performed in accordance with the Guidelines for Experimental Animals established by the Ministry of Science and Technology (Beijing, China). The thigh muscles of Rugao chicken full siblings were collected every two weeks. All samples belonged to the same inbred line but were selected from different families. Twenty-eight individuals at 2 wk, 31 individuals at 4 wk, 23 individuals at 6 wk, 14 individuals at 8 wk, 18 individuals at 10 wk and 19 individuals at 12 wk were killed and sampled. Part of the thigh muscle samples were put in liquid nitrogen and then transferred to a −80 °C freezer. The other part of the thigh muscle samples were kept in an icebox to measure the IMP content.

### Assay of IMP concentration

Fresh and cooling thigh muscle samples were used within 2 h to determine their IMP content. After several sample preparation steps, HPLC (High Performance Liquid Chromatography) was used to analyze the concentration of IMP in the samples, as previously described [18]. The result was analyzed with the one-way ANOVA method in SPSS 18.0.

### Microarray hybridization experiment

Thigh muscle samples of 1 male and 1 female at each time point were selected from full sib samples, and samples were wrapped in 20 kg dry ice in a styrofoam box and transported to Shanghai Biotechnology Co., Ltd (SBC) within 2 days after collection. Samples were directly used for the total RNA extraction experiment and microarray hybridization experiment on 2 chips (1 each for the male and female samples) at each time point as soon as possible. The protocols and kits were provided by SBC. The chicken expression chip was a custom designed product of Agilent (Agilent-027235), which was a single color expression chip that contained 13,378 probes, 8 × 15 K, selected from the commercial microarray of Agilent; the details of all probes could be found in Additional file 1: Table S1. Then, the raw data were normalized by Gene Spring Software 11.5.1 with the Quantile algorithm.

## Analysis of microarray data

### Integrating several metabolism pathways to analyze IMP metabolism pathway based on KEGG database

The normalized microarray data were filtered with Gene Spring Software 11.5.1. By a series of quality control steps, filtered data, which included two chips at each time point as biological duplications, were created with Advanced Analysis operation in Gene Spring. The expression data of samples from 2 wk were used as the control.

The Entrenz gene IDs of 19 IMP genes directly involved in IMP metabolism were obtained from the annotation file and, then, submitted to KEGG (Kyoto Encyclopedia of Genes and Genomes) (http://www.genome.jp/kegg/tool/map_pathway2.html) to retrieve the pathway files from the database to local. These pathway files were loaded into Cytoscape 2.8.2 and were merged into only one network. Some systems biology analysis was carried out on this network via the Network Analyzer plugin.

### Co-expression network analysis

The Pearson correlation coefficients between 19 IMP genes directly involved in IMP metabolism and the other probes on the chip were calculated with Gene Spring Software 11.5.1. Those Genes with Pearson correlation coefficients ≥ 0.85 were visualized by Cytoscape 2.8.2. Finally, the annotation file was imported to determine the attributes of the nodes. All redundant probes were submitted to DAVID and converted to Gene Bank Accession Numbers, which were exported to a file.

### Retrieving the crucial co-expression genes from database of regulatory network of mouse

The accession number file obtained above was used to download the corresponding sequences with a batch download tool from NCBI (National Center of Biotechnology Information). Then, Blastx was use to align the chicken sequences with the mouse protein database. Although the IMP metabolism pathway has been investigated clearly and was found to be particularly conserved in vertebrates, regulatory factors of the IMP metabolism pathway had not been studied. Thus, the homology of protein sequences between chicken and mouse is commonly utilized to identify the corresponding proteins of mouse in chicken, and the regulatory networks of mouse are used to infer the regulatory networks of chicken.

After ID conversion, the Blast results, as accession numbers, were imported to Gene Spring Software 11.5 to construct three different types of networks based on the pathway database of Gene Spring 11.5.1. Some key genes were selected by comparing those three networks. Then, the signal pathways of these genes were further analyzed with KEGG to clarify the regulatory relationship among them and their influence on IMP metabolism.

### Construction regulating network of IMP metabolism

First, we recalculated the Pearson correlation coefficient between the expression pattern of 19 IMP genes directly involved in IMP metabolism and 15 CGs (15 co-expression genes) and IMP content by SPSS 18.0. A network was constructed with Cytoscape 2.8.2 with an edge-weighted spring embedded algorithm, removing edges with a coefficient less than 0.7. Then, the features of this network were statistically analyzed and visualized via the Network Analyzer plugin of Cytoscape. Finally, we integrated the transcriptional regulators network into Fig. 3 and removed the edges among nodes of the same shape with a hierarchical layout using Cytoscape 2.8.2 (Fig. 4).

### Real time quantitative RT-PCR validating the microarray data

We designed a qRT-PCR experiment to validate the microarray expression pattern data. 4 genes were selected from the regulatory network in Fig. 4 for qRT-PCR primer design (Additional file 2: Table S2). The experimental design of qRT-PCR was the same as that for the microarray and used the same RNA samples that were used in the microarray experiment. The relative quantification experiment protocols followed the manuals of PrimeScript® RT Master Mix kit and SYBR® Premix Ex Taq™ II kit (PrimeScript RT Master Mix (Perfect Real Time) DRR036; SYBR Premix Ex Taq II (Tli RNase H Plus) DRR820). A 7900 HT Sequence Detection System was used in this experiment. The 2^-ΔΔCt^ method was applied to analyze the results with Microsoft Excel 2010.

## Results

### IMP variation pattern

The results indicated that the concentration of IMP fluctuated from 2 wk to 12 wk. Males and females were significantly correlated with each other, with a correlation coefficient of 0.822 (Sig. (2-tailed) ≤0.05). Simultaneously, the correlation coefficient of the microarray data between genders illustrated that at each time point, the two sexes had a high level correlation, indicating that their expression profiles were similar (Additional file 2: Table S3).

The amount of total IMP content significantly rose from 2 to 4 wk, reaching 3.8470 at 4 wk. From 4 to 8 wk, the IMP content fluctuated only slightly, and then at 10 wk, it dropped sharply to 3.1643. Ultimately, the IMP content dramatically increased to 4.086 (Table 1 and Additional file 3: Figure S1). To explain the IMP concentration changes, the expression microarrays were used to clarify the different expression spectrums and characteristic expression patterns.

**Table 1:** IMP concentration at each time point from 2 to 12 wk (mg/g)

Groups	Time points					
	2 wk	4 wk	6 wk	8 wk	10 wk	12 wk
Male	2.9073 ± 0.1360^1a^	3.8823 ± 0.1242^bc^	3.4162 ± 0.1740^bc^	3.7006 ± 0.3120^ac^	3.0038 ± 0.2348^ab^	3.8039 ± 0.2969^c^
	(14)^2^	(16)	(11)	(6)	(9)	(9)
Female	2.9822 ± 0.1709^a^	3.8094 ± 0.3132^bc^	3.9376 ± 0.3354^bc^	3.6698 ± 0.3157^ac^	3.3249 ± 0.2548^ab^	4.3400 ± 0.3523^c^
	(14)	(15)	(12)	(8)	(9)	(10)
Total	2.9448 ± 0.1074^a^	3.8470 ± 0.1618^bc^	3.6882 ± 0.1975^bc^	3.6830 ± 0.2162^ac^	3.1643 ± 0.1725^ab^	4.0861 ± 0.2349^c^
	(28)	(31)	(23)	(14)	(18)	(19)

^1^Presentation Manner: Mean ± Standard Error

^2^Individual numbers are in brackets

Containing the same superscript letter (a, b and c) means no significant difference (*P* ≤ 0.05)

### Integrating several relevant metabolism pathways to analyze IMP metabolism pathway based on KEGG database

After normalization and filtering with Gene Spring Software 11.5.1, 1,131 probes with differential expression for at least one time point were chosen. Additionally, 19 IMP genes directly involved in IMP synthesis, decomposition and utilization were chosen. Some of those 19 IMP genes were homologues or isoenzymes. These 19 IMP Genes were *Gart*, *Atic*, *Paics*, *Adsl*, *Prps2*, *Entpd4*, *Nt5c1a*, *Nt5c1b*, *Nme1*, *Prps1*, *Nudt9*, *Ppat*, *Ampd3*, *Entpd5*, *Itpa*, *Nme6*, *Ampd1*, *Adssl1* and *Entpd8* (Additional file 3: Figure S2).

**Fig. 1:**
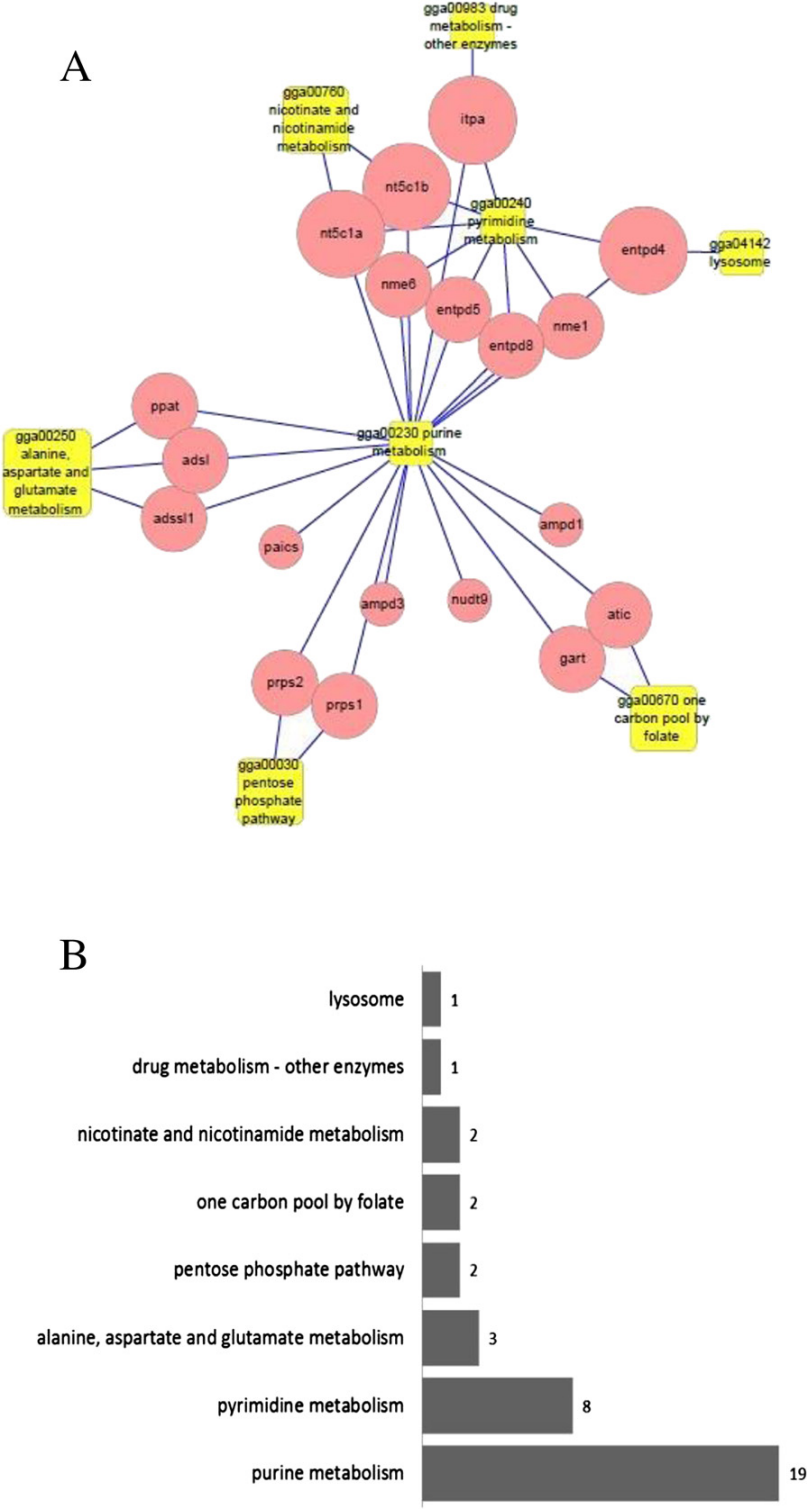
**a** Relation network between the 19 IMP genes and 8 relevant pathways. Round nodes symbolize single genes that were engaged in multiple pathways (larger circles indicate more pathways), and rectangles symbolized single pathways that included multiple genes of the 19 IMP genes (larger rectangles indicate more genes). **b** Histogram of pathways is ordered by the number of included genes of 19 IMP genes

These 19 IMP genes are known to be engaged in 8 metabolic pathways (Fig. 1). The purine metabolism pathway is the central pathway, and the other 7 pathways are affiliate pathways that play an important role in nucleic acid and amino acid metabolism. Thus, these 19 IMP genes or potential genes indirectly involved in the IMP metabolism pathway are all candidate genes for further research.

We detected the key enzymes and compounds that indirectly affected IMP metabolism by identifying those that interacted with the 19 IMP genes from the integrated network (Additional file 3: Figure S3). The degree of enzymes in the network was at least 2. We filtered genes and compounds with total degrees of more than 10.

We identified 7 hub genes with a total degree of more than 10: *Nt5c3*, *Entpd8*, *Nme7*, *769958*, *Itpa*, *Hprt1* and *Rrm1* (Additional file 2: Table S4). The total degrees of L-aspartate,L-glutamate,ADP,GTP,tetrahydrofolate,GMP and IMP were all more than 10 (Additional file 2: Table S5), indicating that these compounds are the primary substrates of nucleic acid metabolism.

### Co-expression network analysis

Next, we calculated the Pearson correlation coefficients [19] of the 19 IMP genes with the other probes with a one way ANOVA corrected *P*-value *P* ≤ 0.05 and fold-change cut-off of 2.0 set as the selection standards to filter the co-expression genes with related coefficients ≥ 0.85. Seven genes were identified that co-expressed with *Ppat*; 34 for *Gart*; 13 for *Paics*; 3 for *Adsl*; 3 for *Atic*; 2 for *Nt5c1b*; 27 for *Nt5c1a*; 23 for *Ampd3*; 10 for *Ampd1*; 16 for *Ipta*; 151 for *Entpd8*; 40 for *Adssl1*; 2 for *Entpd4*; 5 for *Nudt9*; 24 for *Nme1*; 5 for *Nem6*; 5 for *Prps1*; 14 for *Prps2*; and 1 for *Entpd5* (Additional file 3: Figure S4).

A co-expression undirected graph in which the edges symbolized a related coefficient ≥0.85 was constructed with Cytoscape 2.8.2. We found 339 non-redundant nodes from 384 total nodes, including 19 IMP genes. There were 10 sub-networks shown in Additional file 3: Figure S4. *Entpd8* was at the center of the biggest subnet and was a member of the most complex sub-network, which consisted of *Paics*, *Nudt9*, *Adssl1*, *Entpd9*, *Nme1*, *Gart* and *Prps1*, the core nodes of the sub-networks in Additional file 3: Figure S4B. Nets in Additional file 3: Figure S4C had a low complexity with a small number of nodes.

Subsequently, 339 nodes in the co-expression network were analyzed for GO (Gene Ontology) and pathway enrichment. Those 339 nodes contained 204 genes identified by the DAVID website. After using medium classification stringency, those 204 genes were clustered into 5 classes of molecular function, including nucleotide binding, GTPase regulator activity, guanyl-nucleotide exchange factor activity, nucleoside-triphosphatase regulator activity, GTP binding, transcriptional regulator activity and ion binding. By the same token, those 204 genes primarily participated in 14 biologic processes, including the nitrogen compound biosynthetic process, nucleoside metabolic process, glycoprotein biosynthetic process, blood vessel development, ion transport, regulation of cell migration, anterior/posterior pattern formation, hemopoiesis, apoptosis, protein localization, regulation of the transmembrane receptor protein tyrosine kinase signaling pathway, negative regulation of macromolecule biosynthetic processes, positive regulation of nucleobase, nucleoside, nucleotide and nucleic acid metabolic processes, protein amino acid phosphorylation and transcriptional regulation. The results suggest that these 204 genes primarily have regulatory functions at the transcriptional and translational level. Nucleotide metabolism, which includes IMP metabolism, would obviously be regulated by these function molecules or signaling pathways.

The identified co-expression genes were mainly involved in developmental pathways, such as the MAPK, mTOR, TGF-β, PPAR and erbB pathways, indicating that IMP metabolism is regulated by a wide range of genes related to a complicated signal transduction network for organ development.

### Retrieving the crucial co-expression genes from database of regulatory network of mouse

**Fig. 2:**
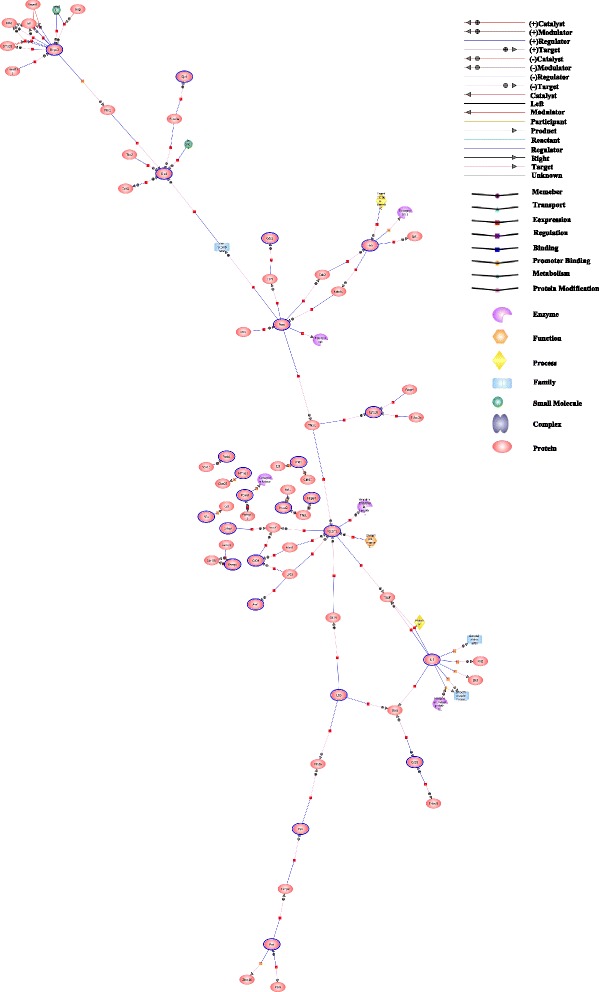
Transcriptional regulators network was constructed with co-expression genes by Gene Spring 11.5.1. The highlight genes were the 15 CGs

In this step, we analyzed 270 probe numbers, and 206 were recognized by Gene Spring 11.5.1. Three networks based on the pathway database of Gene Spring 11.5.1 (Additional file 3: Figure S5A, Figure S5B and Fig. 2) were built. By comparing those three networks, 25 nodes were filtered out. Then, because 10 of these nodes could not be found in the co-expression network, the remaining 15 nodes, *Hspa2*, *Pten*, *Gabpa*, *Bpi*, *Mkl1*, *Srf*, *Cd34*, *Hspa4*, *Etv6*, *Bmpr2*, *Gde 1*, *Igfbp5*, *Cd28*, *Pecam1*, *Gja1*, that appeared in each networks were selected as the candidate genes for further analysis.

The genes in the shortest connection networks always had a pathway link to each other, indicating a much closer relationship between those genes. In the expanded interaction network, these 15 genes not only interacted with a wide range of molecules but were also involved in various biologic process that were primarily linked with transcription, mutation, cell migration, cytokine production, cell differentiation, T cell proliferation, ossification and cell motility or functions mainly including protein kinase activity, phosphatase activity, telomerase activity, RNA binding, protein-glutamine gamma-glutamyl transferase activity, GTPase activity, histone acetyltransferase activity, stearoyl-CoA 9-desaturase activity, insulin-like growth factor binding, ATP binding, heparin binding, 3′-nucleotidase activity, phosphoric monoester hydrolase activity, carboxy-lyase activity and ligase activity.

### Constructing regulatory network of IMP metabolism

However, the relationship between IMP concentration and gene expression patterns was still unclear. Thus, we recalculated the Pearson’s correlation coefficient between the expression pattern of the 19 IMP genes and 15 CGs with IMP content to determine those genes which were highly positive or negative related to IMP content.

**Fig. 3:**
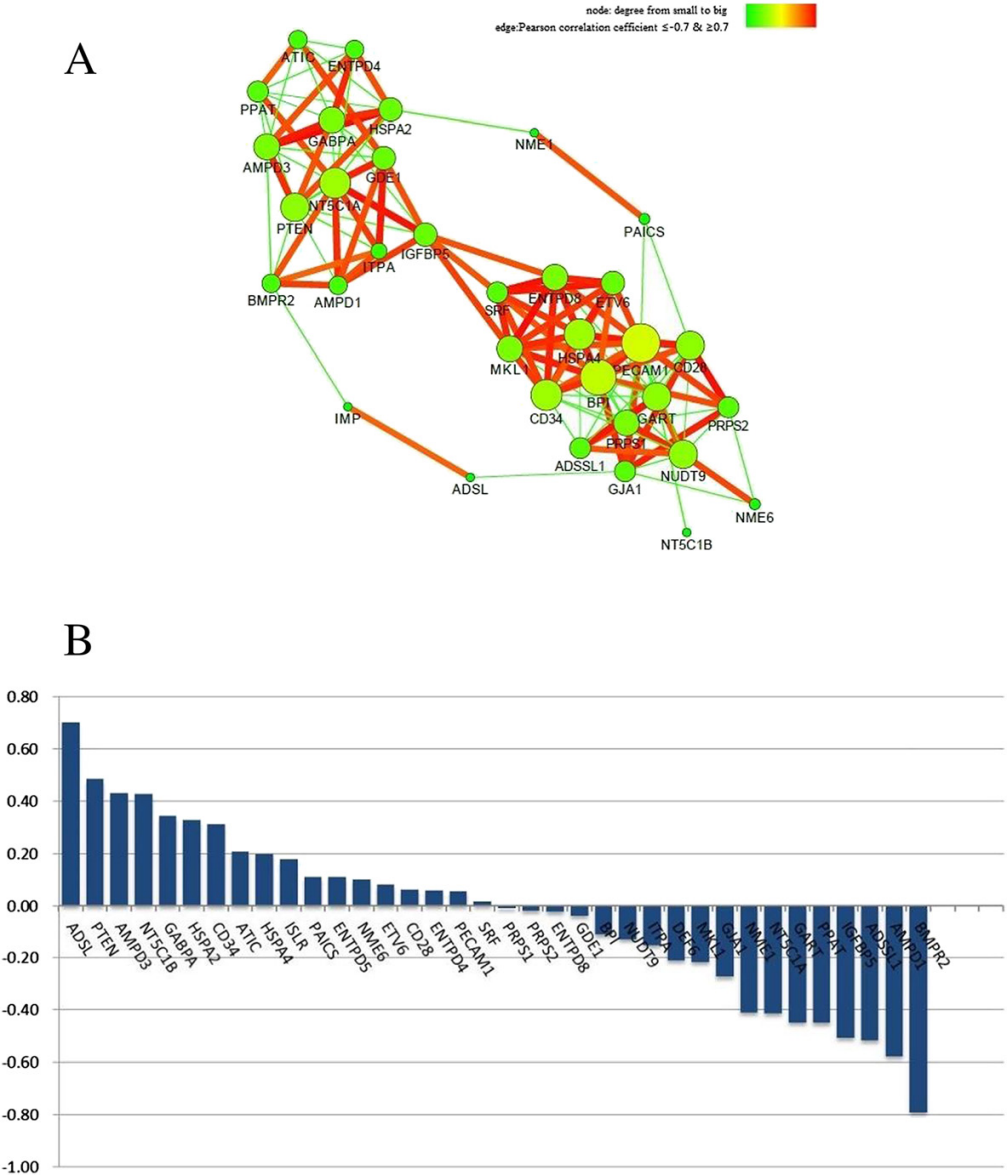
**a** Regulating relation among 19 IMP genes, 15 CGs and IMP content with Pearson correlation coefficient ≥0.7 was visualized by Network Analyzer plugin of Cytoscape. **b** The histogram depicted the relationships between IMP content and 34 genes and was drawn via Microsoft Excel 2010

As seen from Fig. 3, IMP content was positively related to *Adsl* and negatively related to *Bmpr2* above the absolute coefficient value of 0.7; thus, any nodes positively related to *Adsl*, might have an indirect positive regulatory function on IMP metabolism.

Similarly, any nodes negatively related to *Bmpr2*, might have an indirect negative regulatory function on IMP metabolism. As BMPR2 is a negative regulator of muscle growth [20], IMP content might be positively related with muscle growth.

In addition to the IMP node, other nodes gathered and formed two groups, indicating that different groups may be involved in different regulatory patterns. Seven genes directly involved in IMP metabolism of the 13 genes in the left group were chiefly engaged in IMP synthesis and decomposition pathways. Additionally, nine genes directly involved in IMP metabolism of the 17 genes in the right group were mainly involved in other nucleic acid metabolism pathways. *Nme1*, *Paics* and *Adsl* seemed negatively related with these two groups, suggesting that these three genes behave differently from other genes and are worth investigating further.

Next, we focused on merged Figs. 2 and 3. We divided all nodes into 3 groups, 19 IMP genes, 15 CGs and intermediate genes regulated by or regulating co-expression genes. Then, except for the triangle nodes, edges that existed between the diamond nodes group and round nodes group were removed because those relationships did not assist in the determination of the links between the diamond nodes and round nodes. Edges between triangle nodes and round nodes, which had reference support in the mouse regulatory network database, were kept, allowing the potential regulatory network to be constructed.

### Results of qRT-PCR

We choose 4 genes from the analysis results to validate the accuracy of our microarray data. As seen in Table 2, most of the qRT-PCR results had significant correlation coefficients with the results of the microarray experiment, except for *Pecam1*. Although *Pecam1* did not reach the significance level, it still had a comparatively high correlation coefficient, nearly 0.8. Those results indicated that the microarray data were reliable, in spite of the fact that complete assurance of the accuracy of the microarray data would require more qRT-PCR experiments.

**Table 2:** Comparing results of qRT-PCR and results of microarray experiment

Gene symbol	Experiment type	Fold change						Pearson correlation coefficient	Sig.(2-tailed, *P* ≤ 0.05)
		2 wk	4 wk	6 wk	8 wk	10 wk	12 wk		
*Bmpr2*	Results of qRT-PCR	1.00	−1.71	−2.00	−1.53	−1.98	−2.91	0.9248	0.0083
	Results of Microarray	1.00	−2.41	−1.40	−2.19	−1.63	−2.90		
*Prps2*	Results of qRT-PCR	1.00	1.39	−1.97	2.28	1.13	−1.21	0.8633	0.0267
	Results of Microarray	1.00	1.64	−1.35	2.47	1.33	1.05		
*Gja1*	Results of qRT-PCR	1.00	1.29	−1.75	3.31	1.42	−1.09	0.9858	0.0003
	Results of Microarray	1.00	1.01	−1.48	2.72	1.66	−1.37		
*Pecam1*	Results of qRT-PCR	1.00	2.04	−1.08	3.16	1.33	−1.05	0.7994	0.0563
	Results of Microarray	1.00	1.96	1.12	2.87	2.47	1.22		

## Discussion

### Developmental status influenced the variation of IMP content

Different concentrations of IMP may be correlated with different developmental states at the different time points. Although some researchers have reported that IMP content increases during the growth process [12], our result revealed that the IMP content did not consecutively increase from 2 to 12 wk. This is likely because IMP metabolism was considered merely a part of purine metabolism, meaning IMP was an intermediate compound in purine metabolism. Because ATP and GTP are utilized in energy generation, IMP has an affinity for energy metabolic processes [21]. Thus, the efficiency of the de novo synthesis of IMP, the rate of the compensatory pathway of IMP synthesis and the rate of IMP utilization to synthetize other nucleic acids determines the concentration of IMP [22]. The enzymes investigated here, which were involved in these three processes, interacted with more than one substrate. The genes that had a significant effect on the efficiency of IMP metabolism were those that participated in a wide range of metabolic reactions. When chicks grow fast, the IMP synthesize comparatively more slowly than IMP decomposition and utilization, thus, the deposition rate of IMP was not high.

IMP content at 10 wk dropped sharply and then showed subsequent significant increase at 12 wa. Kehua *et al.* [17] found Rugao chicken growth rate slowed down after 7 wk, and shrinked faster from 10 wk to 12 wk than from 8 wk to 10 wk [23]. Laio [24] found that Rugao chicken muscle fiber density of the thigh noticeably decreased from 10 wk to 12 wk. The diameter of the muscle fiber was also increased dramatically from 8 wk to 10 wk but slowed down from 10 wk to 12 wk. Their results were consistent with our result, which suggested that the increase of the diameter from 8 wk to 10 wk probably promoted the metabolism rate of the IMP. Subsequently, the increasing rate of the diameter of muscle fiber decreased and fiber density reduced faster than from 8 wk of age to 10 wk, which provide a time window for the accumulation of the IMP before slaughter.

Some substrates identified were involved in a wide range of reactions based on the KEGG database, including IMP, L-aspartate,L-glutamate,ADP,GTP,tetrahydrofolate and GMP. Especially, the total degrees of L-aspartate and L-glutamate were more than other compounds, which indicates that many substrates participating in amino acid metabolism pathways are converted to these two amino acids. And these two compounds may facilitate IMP synthesis in muscle cells.

When cells proliferate quickly, the needs of genome and transcriptome synthesis are increased. IMP metabolism is one part of nucleic acid metabolism, and the variation of nucleic acid metabolism influence IMP metabolism as well [25]. Thus, the developmental pattern of muscle significantly influences the synthesis and decomposition of IMP. It was reported that increased ATP or ADP content depressed the synthesis of IMP; however, IMP can also be synthesized by a salvage pathway. The content of IMP is a dependent variable that is decided by several independent variables involved in the complex metabolic and regulatory network.

### IMP synthesis was influenced by a complex regulatory network through *Entpd8*

Subsequently, we focused on the co-expression genes of a group of enzymes directly participating in IMP metabolism. The high correlation coefficient illustrated that the complexity of the sub-networks reflected the assembling trend of nodes belonged to similar expression patterns. There were some nodes shared by several core genes in Additional file 3: Figure S4B and S4C, having a correlation coefficient value simultaneously higher than the standard value with those core genes, those genes might be involved in the regulation of IMP synthesis and catabolism. Furthermore, we noticed that these co-expression genes located in the crucial pathway of growth and development.

There was a type of nodes positively connected to *Entpd8* described by participation in the salvage synthesis pathway by catalyzing ITP into IDP and IDP into IMP. *Entpd8* had a large numbers of co-expressed genes, suggesting that *Entpd8* is widely regulated and many of its regulatory genes are correlated with IMP metabolism. Some of the potential regulatory genes included in these 151 co-expressed genes were transcriptional regulatory factors that directly regulate the expression of *Entpd8* or indirectly regulate the expression of other regulatory factors. Thus, our results indicate that IMP synthesis is influenced by a complex regulatory network through *Entpd8*.

### 15 crucial co-expression genes mainly were involved in muscle development

We then utilized GeneSpring to construct three different types of networks. After comparing them with each other, we identified 15 crucial co-expression genes. The results illustrated that as an accessary substance of nucleic acid metabolism, IMP content in thigh muscles varies with a series of processes and molecular activities. The regulatory relationships among these 15 genes in a transcriptional regulatory network was clearly presented, which were supported by references, such as *Bmpr2* regulating the expression of *Msx1* [26]. These 15 genes had a high potential to be regulators of expression of the enzymes of IMP metabolism (Additional file 2: Table S6).

We focused on these 15 genes and tried to find their positions and functions in the regulatory networks. Fifteen CGs were submitted to KEGG to determine the pathway they participated in (Additional file 2: Table S7). As a result, the 15 CGs were found to be involved in 20 pathways that influenced proliferation, differentiation, immunity response and adhesion of muscle cells. Thus, some genes had positive effects on muscle differentiation and proliferation.

Firstly, the SRF protein is closely related with the muscle development process and binds the promoters of some genes involved in muscle proliferation, such as genes of the family of smooth muscle myosin light chain kinases and skeletal alpha actin. Aline G *et al.* found that SRF regulated function of satellite cells and supported muscle growth [27].

Recently, it was reported in chicken that from day 18 of the embryonic period to 43 days after hatching, the expression level of *Pten* decreased dramatically in muscle tissue [28]. The research of Allander *et al.* suggested that the IGFBP5 protein of chicken has a similar function to *Pten*, and its function, which particularly affects the differentiation of muscle, is conserved in all vertebrate species [29].

GABPA, which mainly accumulates in the neuromuscular junction, regulates expression of AChR and is modulated by NRG-1 via phosphorylation [30]. Brzoska *et al.* reported that CD34, which is mainly expressed in specific stem cells, is mobilized by positive impact of SDF-1 to help muscle regeneration [31]. Xu *et al.* found that *Hspa4*, which was one of our 15 identified CGs, in the breast muscle of duck had high expression levels at 8 wk and showed a tendency to increase with age (determined from microarray expression data) [32]. Johnstone *et al.* identified MAPK-phosphorylated GJA1 (cx43) as a new interacting partner of cyclin E in VSMC (vascular smooth muscle cell) and showed that this interaction is critical for VSMC proliferation [33].

### Constructed regulation network revealed some new regulators influencing IMP metabolism

Keeping sufficient potential candidate genes could avoid omitting crucial modulation factors. Thus, we used all 34 nodes to construct the regulatory network. The dashed lines indicate CGs that regulated the 19 IMP genes, their real relationships were not clear because no evidence was found to explain whether these 15 CGs could interact with those 19 IMP genes or regulate the expression of those 19 IMP Genes. We assumed that these 15 CGs had at least a potential ability to influence the expression of those 19 IMP genes. According to this hypothesis, we could determine the regulatory factors and the regulatory order.

**Fig. 4:**
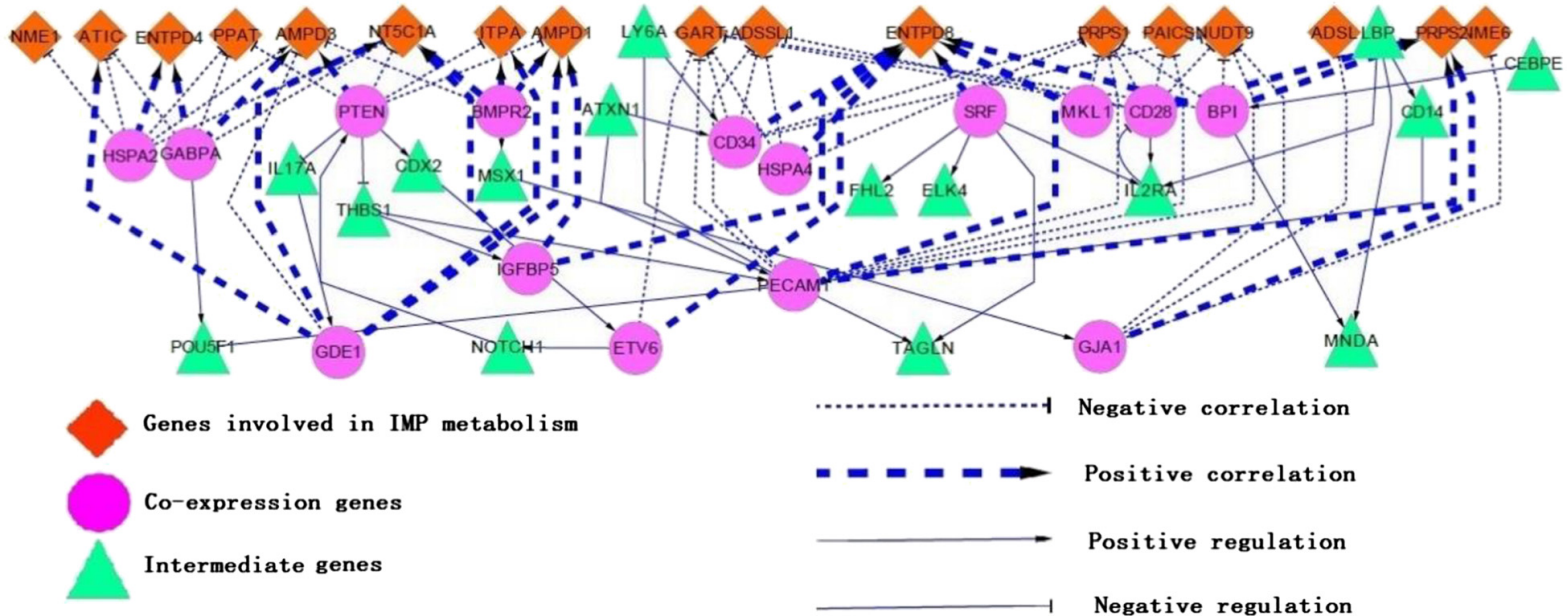
Integrated regulatory graph was constructed as a prediction for the regulatory pathway of IMP metabolism

Interestingly, among those 34 genes identified, only one gene, *Bmpr2*, was reported negatively regulating muscle development [20], and we found it also is negatively correlated with IMP content (Fig. 3B). According to further correlation coefficient analysis, *Bmpr2* was negatively correlated with *Ampd3* and was positively correlated with *Itpa*, *Nt5c1a* and *Ampd1*, indicating that *Bmpr2* might positively modulate IMP decomposition (Fig. 3A). Especially, through the *Msx1* activation of *Gja1*, *Bmpr2* might indirectly and negatively modulate *Nme6*, *Nudt9* and *Adsl*, which widely participate in the metabolism of different types of nucleic acids (Fig. 4). *Prps2* catalyzes the conversion of D-ribose 5-phosphate into 5-phospho-alpha-D-ribose 1-diphosphate, which is commonly considered the rate-limiting step of IMP de novo synthesis and to affect dNTP-pool deposition, which influences cell proliferation [34]. We found that *Prps2* may be positively regulated by *Gja1*. However, 5-phospho-alpha-D-ribose 1-diphosphate did not play a unique role in purine metabolism, but was involved in the pentose phosphate pathway. Regardless, *Gja1* has a negative relation with IMP content. That is to say, *Bmpr2* mainly function is to boost the pentose phosphate pathway and decomposition of IMP, and at the same time represses the IMP utilization pathway via the TGFβ-TAK1-MAPK signaling pathway.

PTEN was also involved in the TGFβ-TAK1-MAPK signaling pathway, according to the KEGG database, and was negatively connected with those genes involved in IMP decomposition according to Fig. 4, which suggested it was a negative regulator of IMP decomposition. However, through the *Thbs1* positive expression regulation of *Pecam1*, *Pten* indirectly up regulates *Prps2*.

Some genes and pathways regulating IMP were likely to be missed by our study, but we also identified many genes and pathways that were highly enriched and had different impact on muscle cell differentiation and proliferation. As a part of nucleic acid metabolism, IMP metabolism was also affected by these genes and pathways. To sum up, we found some regulatory factors and constructed a model of the regulation of IMP metabolism, but specific regulatory mechanisms require further study.

## Conclusions

According to the results of the co-expression analysis, 15 crucial co-expression genes that showed differential expression for at least one time point were identified as crucial regulators of IMP metabolism. Based on this study, a hypothetical regulatory network, which will be an important reference, was constructed primarily via bioinformatics methods. The results of this work provided a fundamental material and orientation for the breeding of good flavor in poultry meat.

## Additional files

Additional file 1: Table S1. The annotation information of the custom designed microarray. Additional file 2: Table S2. Primers used in real time quantitative RT-PCR analysis. **Table S3.** Correlation coefficient of chips between different genders at the same time point. **Table S4.** Genes with total degree ≥10 in integrated network. **Table S5.** Compounds with Total Degree ≥ 10 in Integrated Network. **Table S6** Pearson coefficients between 15 crucial co-expression genes and relevant genes from the 19 genes. **Table S7.** Pathway Distributions of Co-expression Genes. Additional file 3: Figure S1. Histogram chart of chicken thigh muscle IMP concentration at different time points containing the same letter means no significant difference (*P* ≤ 0.05). **Figure S2.** Location of 19 genes which directly involved in purine metabolism on the map of nucleic metabolism. **Figure S3.** Integrated network of 8 IMP relevant pathways. **Figure S4.** Visualization of co-expression network there were 10 sub-networks. Sub-networks of A and B were bigger than sub-networks in C. **Figure S5.** A: the shortest connection networks; B: the expanded interaction network. Both were generated via Genespring 11.5.1.
