# Supplementary material for: Mining the key regulatory genes of chicken inosine 5′-monophosphate metabolism based on time series microarray data
Source: J Anim Sci Biotechnol. 2015 May 23;6(1):21. doi: 10.1186/s40104-015-0022-3 (PMC4464707; doi:10.1186/s40104-015-0022-3)
Supplement: Additional file 2: Table S2. — Primers used in real time quantitative RT-PCR analysis. Table S3. Correlation coefficient of chips between different genders at the same time point. Table S4. Genes with total degree ≥10 in integrated network. Table S5. Compounds with Total Degree ≥ 10 in Integrated Network. Table S6 Pearson coefficients between 15 crucial co-expression genes and relevant genes from the 19 genes. Table S7. Pathway Distributions of Co-expression Genes. [file 40104_2015_22_MOESM2_ESM.docx]

Mining the key regulatory genes of chicken inosine 5'-monophosphate metabolism based on time series microarray data

Teng Ma^1^, Lu Xu^1^, Hongzhi Wang^1^, Jing Chen^1^, Lu Liu^1^, Guobin Chang^1^*, Guohong Chen^1^*

1. Animal Genetic Resources Laboratory, College of Animal Science and Technology, Yangzhou University.

## Supplement Tables

**Table S2** Primers used in real time quantitative RT-PCR analysis

**Table S3** Correlation coefficient of chips between different genders at the same time point

**Table S4** Genes with total degree ≥10 in integrated network.

**Table S5** Compounds with Total Degree≥10 in Integrated Network.

**Table S6** Pearson coefficients between 15 crucial co-expression genes and relevant genes from the 19 genes.

**Table S7** Pathway Distributions of Co-expression Genes.

Table S2 Primers used in real time quantitative RT-PCR analysis

| Gene Symbol | Probe No. | Direction | Primer Sequence（5′-3′） |
| --- | --- | --- | --- |
| PECAM1 | A_87_P022179 | Forward Primer | GGCAGAACATAGCTCAGCACAA |
|  |  | Reverse Primer | GCACAGGGAGTTCAGCACAA |
| GJA1 | A_87_P009369 | Forward Primer | GGCAGCACCATCTCCAACTC |
|  |  | Reverse Primer | TTTTCGTGTTCTGGTGCTCATC |
| PRPS2 | A_87_P017474 | Forward Primer | AAATGAAACACTGCCCCAAAAT |
|  |  | Reverse Primer | GATACAGATTCACCGTTGTGTGTTC |
| BMPR2 | A_87_P008740 | Forward Primer | GATGAGCATGAACCATTGTTGAG |
|  |  | Reverse Primer | AGGCGGTCCAGAACACCTT |
| GAPDH | Internal Reference | Forward Primer | AAGCAGGACCCTTTGTTGGA |
|  |  | Reverse Primer | ACTGGCCTCTCACTGCAGGAT |

Table S3 Correlation coefficient of chips between different genders at the same time point

| Age | 2 weeks of age | 4 weeks of age | 6 weeks of age | 8 weeks of age | 10 weeks of age | 12 weeks of age |
| --- | --- | --- | --- | --- | --- | --- |
| Correlation Coefficient of different genders | 0.9770 | 0.9397 | 0.9514 | 0.9723 | 0.9755 | 0.9384 |

Table S4 Genes with total degree ≥10 in integrated network.

| Gene Name | In-Degree | | Out-Degree | | Total |  |
| --- | --- | --- | --- | --- | --- | --- |
| Nt5c3 | | | 9 | | 15 | 24 |
| Entpd8 | | | 11 | | 12 | 23 |
| Nme7 | | | 0 | | 22 | 22 |
| 769958 | | | 2 | | 15 | 17 |
| Itpa | | | 8 | | 8 | 16 |
| Hprt1 | | | 3 | | 9 | 12 |
| Rrm1 | | | 5 | | 5 | 10 |

Table S5 Compounds with Total Degree≥10 in Integrated Network.

| Compound Name | In-Degree | Out-Degree | Total |
| --- | --- | --- | --- |
| L-Aspartate | 11 | 3 | 14 |
| L-Glutamate | 11 | 2 | 13 |
| ADP | 8 | 2 | 10 |
| GTP | 5 | 5 | 10 |
| Tetrahydrofolate | 9 | 1 | 10 |
| GMP | 8 | 2 | 10 |
| IMP | 7 | 3 | 10 |

Table S6 Pearson coefficients between 15 crucial co-expression genes and relevant genes from the 19 genes.

| Relevant Genes From The 19 Genes | | Probes of Relevant Genes From The 19 Genes | Crucial Co-Expression Genes | Probes of Crucial Co-Expression Genes | Pearson Correlation Coefficient |
| --- | --- | --- | --- | --- | --- |
| AMPD3 | A_87_P024769 | HSPA2 | A_87_P009269 | 0.98 |  |
| AMPD3 | A_87_P024769 | PTEN | A_87_P004080 | 0.92 |  |
| AMPD3 | A_87_P024769 | GABPA | A_87_P017387 | 0.88 |  |
| ENTPD8 | A_87_P017232 | BPI | A_87_P024333 | 0.97 |  |
| ENTPD8 | A_87_P017232 | MKL1 | A_87_P029682 | 0.96 |  |
| ENTPD8 | A_87_P017232 | SRF | A_87_P009061 | 0.95 |  |
| ENTPD8 | A_87_P017232 | CD34 | A_87_P021665 | 0.93 |  |
| ENTPD8 | A_87_P017232 | HSPA4 | A_87_P023938 | 0.93 |  |
| ENTPD8 | A_87_P017232 | ETV6 | A_87_P023953 | 0.88 |  |
| GART | A_87_P129698 | BMPR2 | A_87_P008740 | 0.90 |  |
| ITPA | A_87_P028831 | GDE1 | A_87_P024214 | 0.90 |  |
| NT5C1A | A_87_P027849 | IGFBP5 | A_87_P020259 | 0.95 |  |
| NT5C1A | A_87_P027849 | GDE1 | A_87_P024214 | 0.88 |  |
| PRPS2 | A_87_P017474 | CD28 | A_87_P008861 | 0.93 |  |
| PRPS2 | A_87_P017474 | PECAM1 | A_87_P022179 | 0.87 |  |
| PRPS2 | A_87_P017474 | GJA1 | A_87_P009369 | 0.86 |  |

Table S7 Pathway Distributions of Co-expression Genes.

| Pathway | Gene ID |
| --- | --- |
| Inositol phosphate metabolism - Gallus gallus (1) | PTEN |
| Spliceosome - Gallus gallus (1) | HSPA2 |
| Protein processing in endoplasmic reticulum - Gallus gallus (1) | HSPA2 |
| Ubiquitin mediated proteolysis - Gallus gallus (1) | PML |
| MAPK signaling pathway - Gallus gallus (2) | SRF |
|  | HSPA2 |
| TGF-beta signaling pathway - Gallus gallus (1) | BMPR2 |
| Phosphatidylinositol signaling system - Gallus gallus (1) | PTEN |
| mTOR signaling pathway - Gallus gallus (1) | PTEN |
| Cytokine-cytokine receptor interaction - Gallus gallus (1) | BMPR2 |
| Cell adhesion molecules (CAMs) - Gallus gallus (3) | CD28 |
|  | CD34 |
|  | PECAM1 |
| Endocytosis - Gallus gallus (2) | PML |
|  | HSPA2 |
| p53 signaling pathway - Gallus gallus (1) | PTEN |
| Focal adhesion - Gallus gallus (1) | PTEN |
| Tight junction - Gallus gallus (1) | PTEN |
| Gap junction - Gallus gallus (1) | GJA1 |
| Intestinal immune network for IgA production - Gallus gallus (1) | CD28 |
| Dorso-ventral axis formation - Gallus gallus (1) | ETV6 |
| Influenza A - Gallus gallus (2) | PML |
|  | HSPA2 |
| Hepatitis B - Gallus gallus (1) | PTEN |
| Herpes simplex infection - Gallus gallus (1) | PML |
