# Supplementary material for: Mining the key regulatory genes of chicken inosine 5′-monophosphate metabolism based on time series microarray data
Source: J Anim Sci Biotechnol. 2015 May 23;6(1):21. doi: 10.1186/s40104-015-0022-3 (PMC4464707; doi:10.1186/s40104-015-0022-3)
Supplement: Additional file 3: Figure S1. — Histogram chart of chicken thigh muscle IMP concentration at different time points containing the same letter means no significant difference (P ≤ 0.05). Figure S2. Location of 19 genes which directly involved in purine metabolism on the map of nucleic metabolism. Figure S3. Integrated network of 8 IMP relevant pathways. Figure S4. Visualization of co-expression network there were 10 sub-networks. Sub-networks of A and B were bigger than sub-networks in C. Figure S5. A: the shortest connection networks; B: the expanded interaction network. Both were generated via Genespring 11.5.1. [file 40104_2015_22_MOESM3_ESM.docx]

Mining the key regulatory genes of chicken inosine 5'-monophosphate metabolism based on time series microarray data

Teng Ma^1^, Lu Xu^1^, Hongzhi Wang^1^, Jing Chen^1^, Lu Liu^1^, Guobin Chang^1^*, Guohong Chen^1^*

1. Animal Genetic Resources Laboratory, College of Animal Science and Technology, Yangzhou University.

## Supplementary Figures

**FigureS1** Histogram chart of chicken thigh muscle IMP concentration at different time points Containing the same letter means no significant difference (p-value ≤0.05)..

**Figure S2** Location of 19 genes which directly involved in purine metabolism on the map of nucleic metabolism.

**Figure S3** Integrated network of 8 IMP relevant pathways.

**Figure S4** Visualization of co-expression network there were 10 sub-networks. Sub-networks of A and B were bigger than sub-networks in C.

**Figure S5** A: the shortest connection networks; B: the expanded interaction network. Both were generated via Genespring 11.5.1.


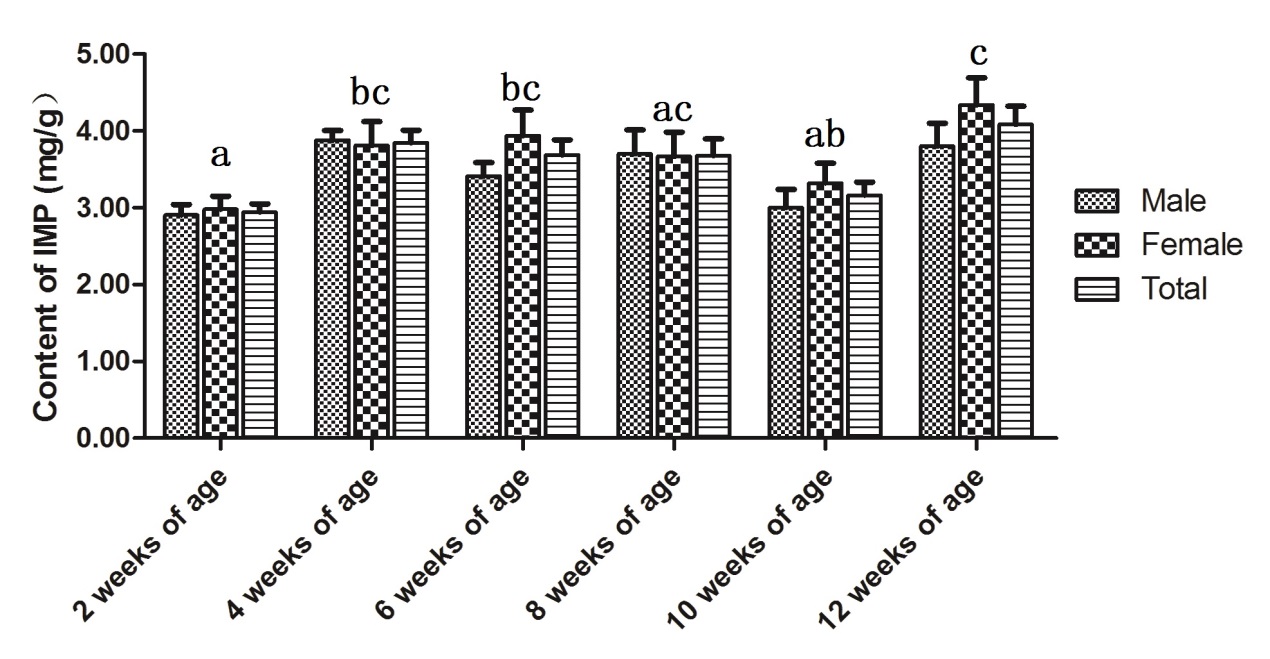


FigureS1 Histogram chart of chicken thigh muscle IMP concentration at different time points Containing the same letter means no significant difference (p-value ≤0.05)..


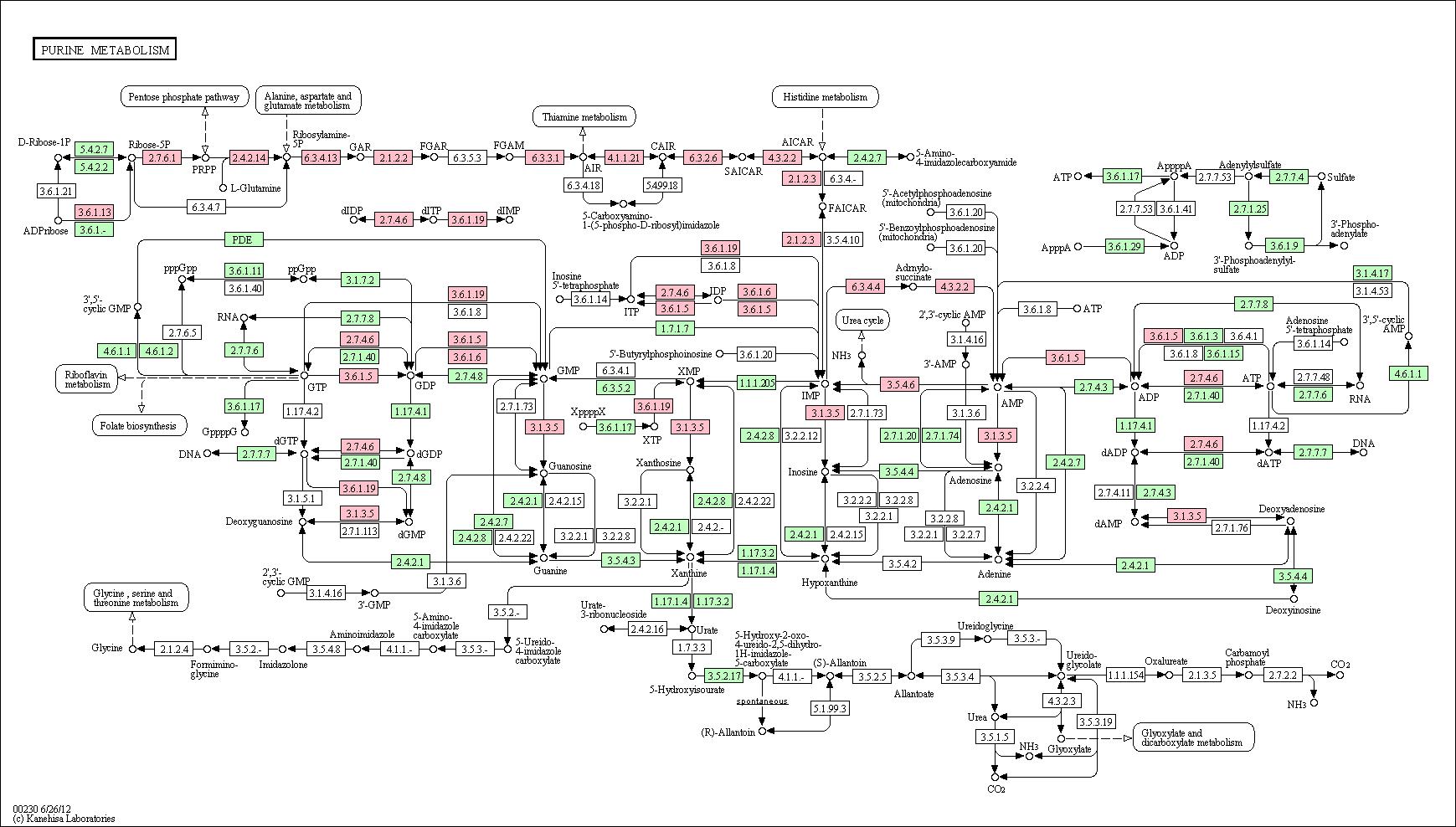


Figure S2 Location of 19 genes which directly involved in purine metabolism on the map of nucleic metabolism.


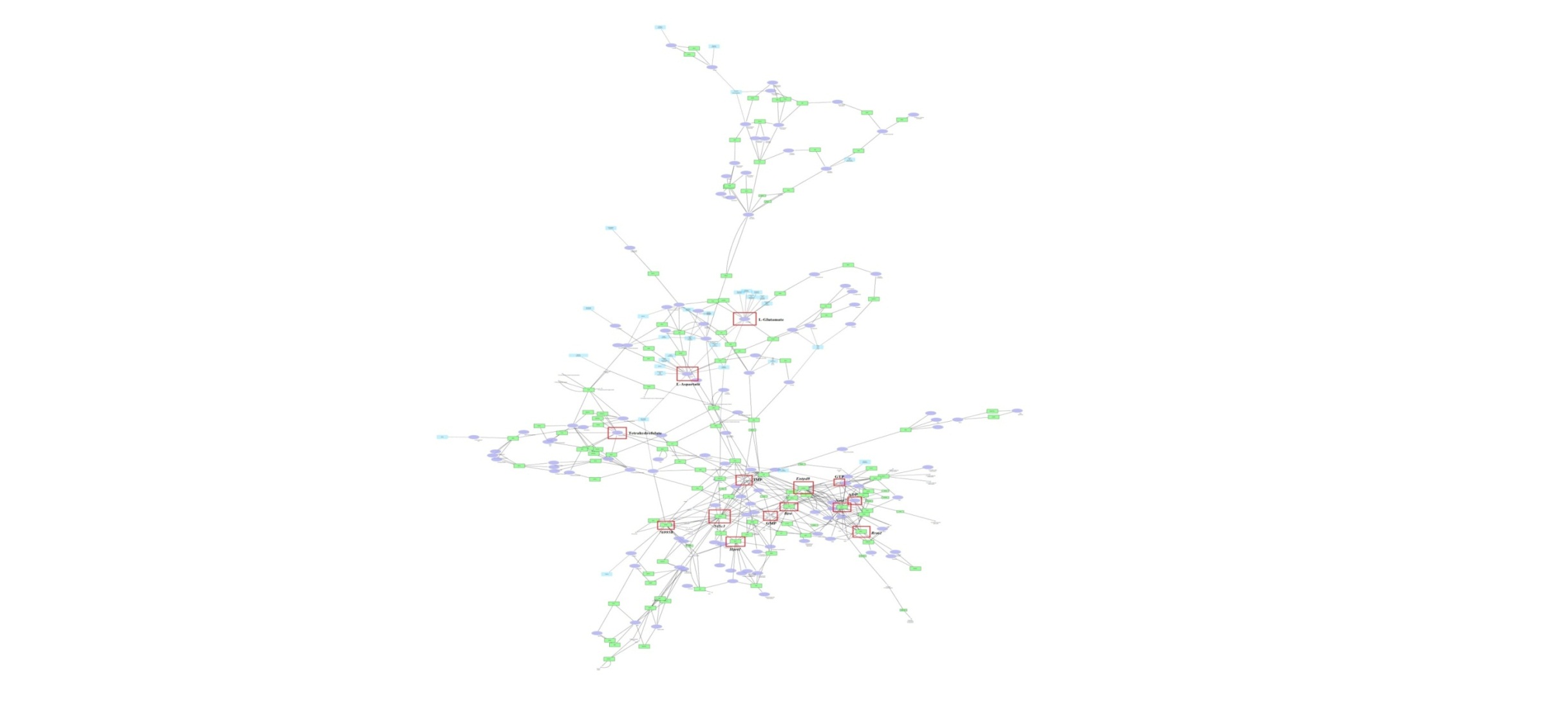


Figure S3 Integrated network of 8 IMP relevant pathways.


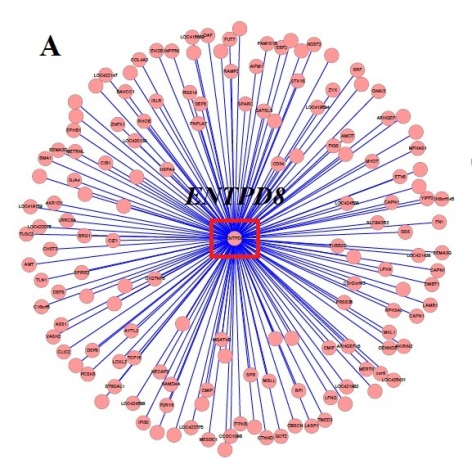

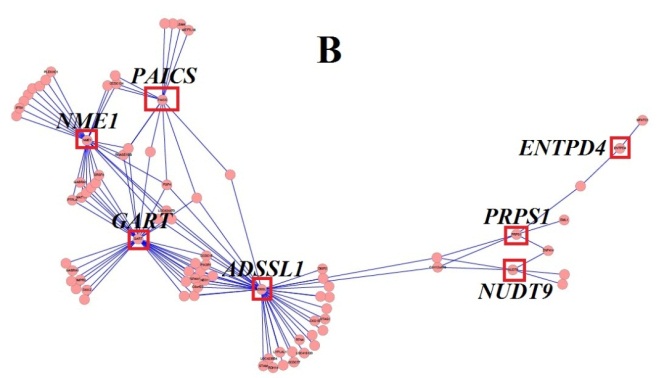


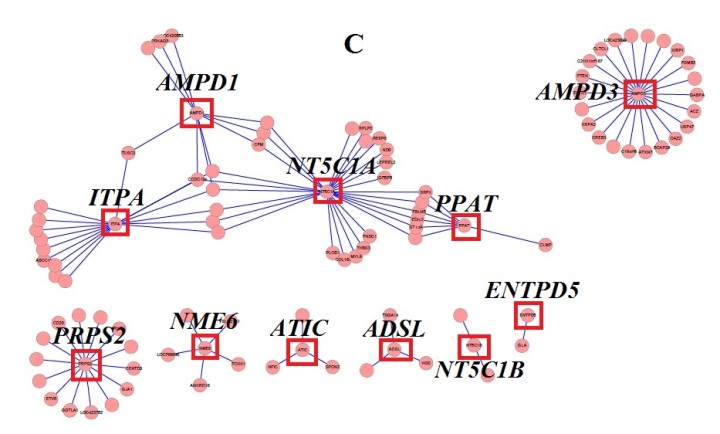


Figure S4 Visualization of co-expression network there were 10 sub-networks. Sub-networks of A and B were bigger than sub-networks in C.


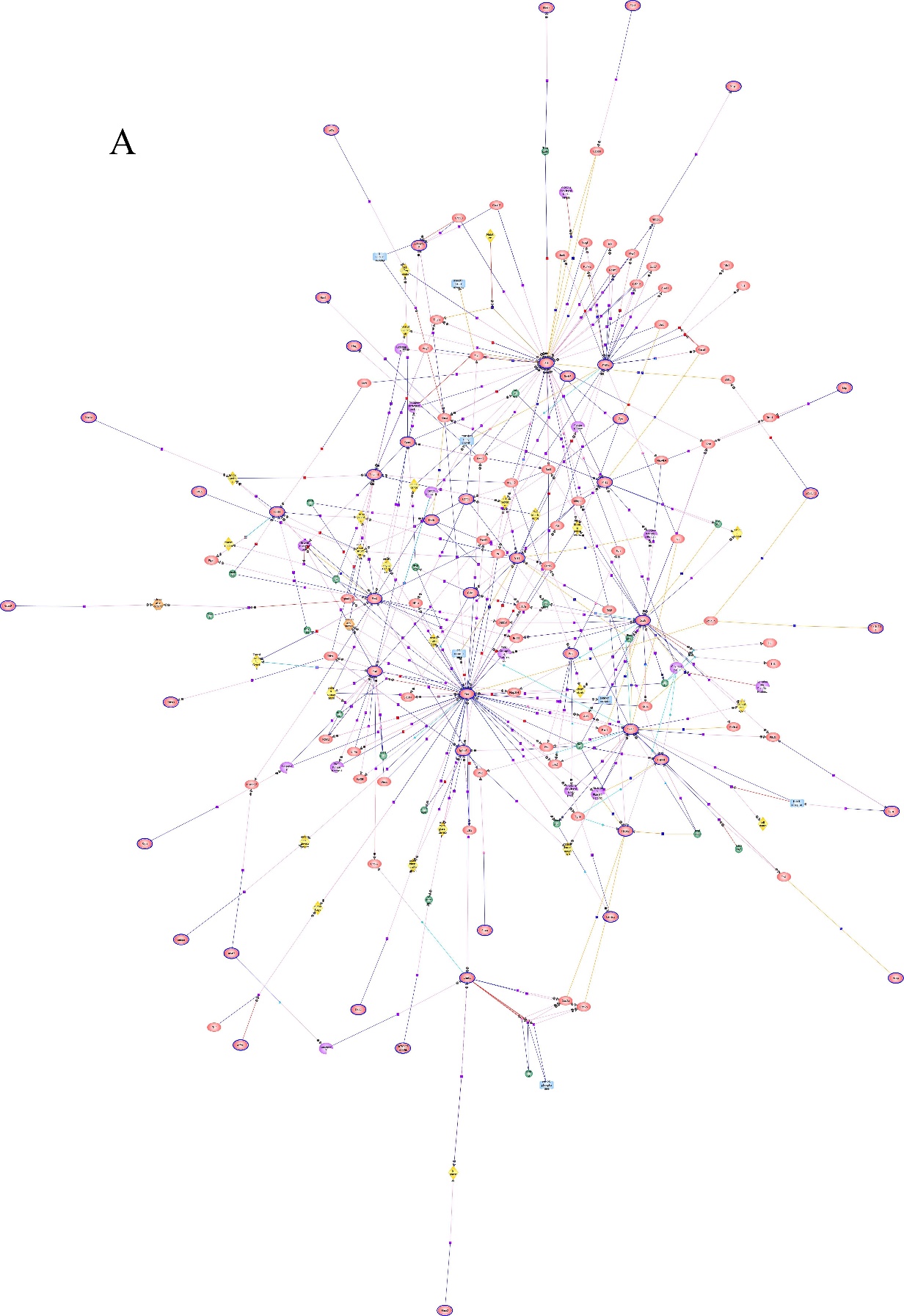


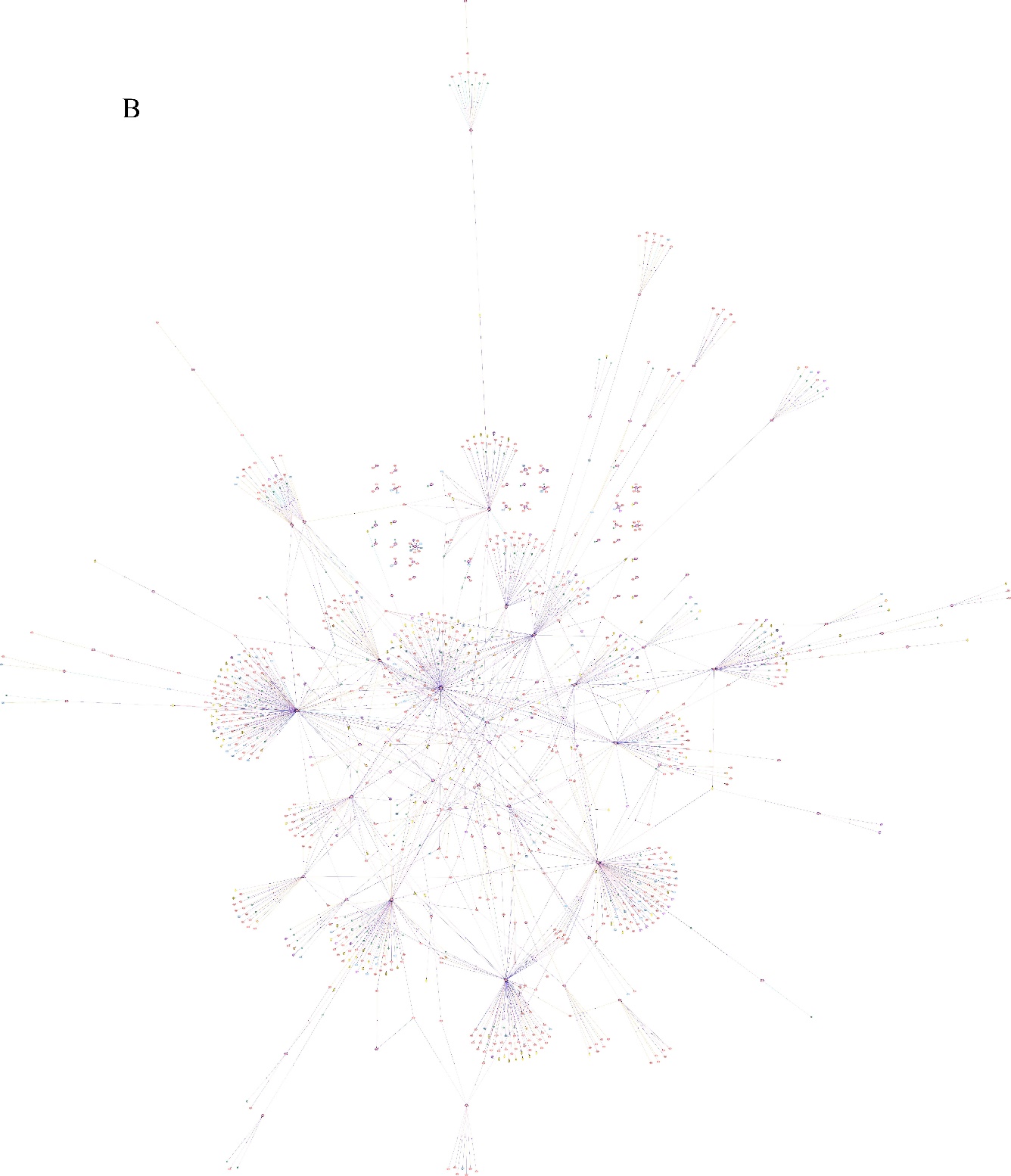


Figure S5 A: the shortest connection networks; B: the expanded interaction network. Both were generated via Genespring 11.5.1.
